# Thymoquinone-Mediated Modulation of Toll-like Receptors and Pluripotency Factors in Gingival Mesenchymal Stem/Progenitor Cells

**DOI:** 10.3390/cells11091452

**Published:** 2022-04-25

**Authors:** Mohamed Mekhemar, Johannes Tölle, Yasmine Hassan, Christof Dörfer, Karim Fawzy El-Sayed

**Affiliations:** 1 Clinic for Conservative Dentistry and Periodontology, School of Dental Medicine, Christian-Albrecht’s University, 24105 Kiel, Germany; toelle.johannes@t-online.de (J.T.); yasminemounir87@gmail.com (Y.H.); doerfer@konspar.uni-kiel.de (C.D.); karim.fawzy@gmail.com (K.F.E.-S.); 2 Oral Medicine and Periodontology Department, Faculty of Oral and Dental Medicine, Cairo University, Cairo 12613, Egypt

**Keywords:** thymoquinone, Toll-like receptors, gingiva, stem cells, stemness, pluripotency

## Abstract

Thymoquinone (TQ), the key active component of *Nigella sativa* (NS), demonstrates very promising biomedical anti-inflammatory, antioxidant, antimicrobial and anticancer properties. Several investigations have inspected the modulative activities of TQ on different stem/progenitor cell types, but its possible role in the regulation of gingival mesenchymal stem/progenitor cells (G-MSCs) has not yet been characterized. For the first time, this study investigates the effects of TQ on G-MSCs’ stemness and Toll-like receptor expression profiles. G-MSCs (*n* = 5) were isolated, sorted via anti-STRO-1 antibodies and then disseminated on cell culture dishes to create colony-forming units (CFUs), and their stem/progenitor cell attributes were characterized. TQ stimulation of the G-MSCs was performed, followed by an examination of the expression of pluripotency-related factors using RT-PCR and the expression profiles of TLRs 1–10 using flowcytometry, and they were compared to a non-stimulated control group. The G-MSCs presented all the predefined stem/progenitor cells’ features. The TQ-activated G-MSCs displayed significantly higher expressions of TLR3 and NANOG with a significantly reduced expression of TLR1 (*p* < 0.05, Wilcoxon signed-rank test). TQ-mediated stimulation preserves G-MSCs’ pluripotency and facilitates a cellular shift into an immunocompetent-differentiating phenotype through increased TLR3 expression. This characteristic modulation might impact the potential therapeutic applications of G-MSCs.

## 1. Introduction

In periodontal disease and during the subsequent healing process, gingival mesenchymal stem/progenitor cells (G-MSCs) represent the vital cellular components involved in the repair of damaged tissues [1,2]. Recent studies explained the remarkable regenerative and immunomodulatory capacity of G-MSCs in vivo and in vitro, representing a promising therapeutic modality of different inflammatory or degenerative conditions [1,3,4,5,6]. Toll-like receptors (TLRs) link innate and acquired branches of immunity, primarily detecting pathogen-associated and damage-associated molecular patterns. Ten extra- and intra-cellular TLRs were described on different types of stem/progenitor cells [1,3,7,8,9,10]. These studies clarified that the specific patterns of TLR expression can differ according to the MSCs’ tissue of origin, as well as microenvironmental factors, such as inflammation or external stimulants. Furthermore, these investigations displayed a specific profile of TLRs in G-MSCs, with a notable effect on several cellular functions and biological activities [1,3,7].

**Figure 1:**
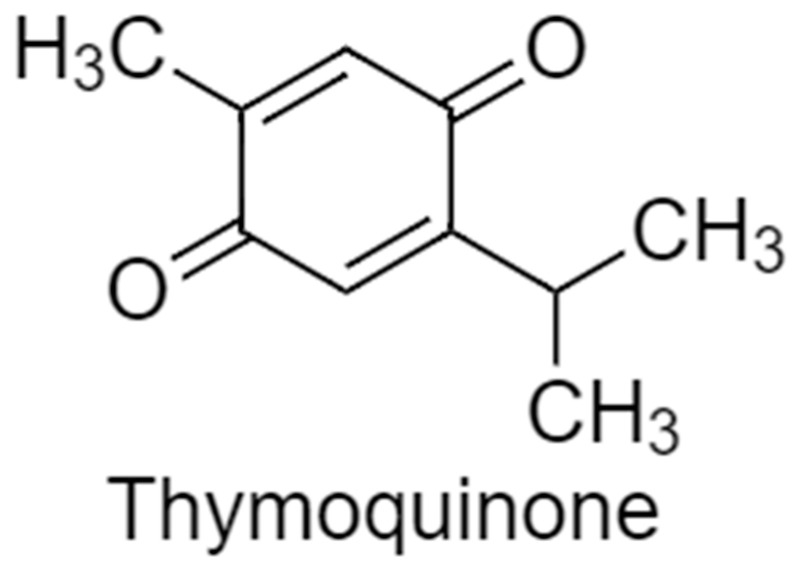
Chemical structure of *thymoquinone*.

Recently, the use of therapeutic herbs in various therapeutic approaches has gained extensive attention due to the reported positive outcomes with minimal, if any, side effects [11]. According to the World Health Organization (WHO), two-thirds of the world’s population has access to herbal medicines [12]. Thus, the WHO has encouraged medical communities to integrate therapeutic plants into their treatment protocols in order to increase their effectiveness and decrease potential costs [11,12]. Among their discrepant uses within different branches of medicine and biological sciences, natural compounds exhibit remarkable abilities to modulate immune receptors, migration, proliferation, cell fate determination and the stemness of different kinds of MSCs [13,14,15]. *Nigella sativa*, a “herb of blessing” established in Islamic medicine and other traditional therapies, also known as black cumin, black seed, or habbatul barakah, is among the most effective evidence-based herbal remedies with various medicinal assets [11]. Throughout history, it was employed in multiple therapeutic settings due to its active constituent thymoquinone (TQ), with the molecular formula C_10_H_12_O_2_ and a molar mass of 164.20 g/mol [11] (Figure 1). The concentration of TQ in *Nigella sativa* oil has been reported to be between 18 and 25 µg/mL. In thermogravimetric examination, the thermal breakdown of TQ starts at 65 °C up to 213 °C. Earlier investigations have reported that TQ is a member of the monoterpene natural complexes and shows keto-enol tautomerism [11]. Explicitly, the keto configuration is the chief structure involved in the beneficial properties of TQ. Moreover, TQ is mostly unstable in aqueous solutions, predominantly at alkaline pH values, and possess severe light sensitivity [11]. Multiple investigations demonstrated various effects of TQ on MSCs’ stemness and pluripotency factors in vivo and in vitro, in addition to their immunomodulatory function and healing-promoting attributes [11,16,17,18,19]. To date, TQ-induced effects on TLR expression and stemness-related pluripotency factors of G-MSCs have not been explored. The aim of the present study was, for the first time, to describe such TQ-mediated modulation in G-MSCs.

## 2. Materials and Methods

### 2.1. G-MSCs’ Isolation and Culture 

G-MSCs’ isolation and culture were performed as previously described [1]. Samples of the free gingiva were isolated surgically from five systemically healthy individuals (*n* = 5; Table 1).

The samples were collected at the Clinic of Christian-Albrecht University, Kiel, Germany, after obtaining informed consent from the individuals (Ethical Committee IRB approval D513/17). After de-epithelization, the samples were cultured in Minimum Essential Medium Eagle, Alpha Modification (α-MEM; Sigma-Aldrich GmbH, St. Louis, MS, USA), supplemented with 100 µg/mL streptomycin, 2 mM l-glutamine and 100 U/mL penicillin, and 1% amphotericin (Biochrom AG, Berlin, Germany). The samples were left in Petri dishes (Sarstedt AG, Nümbrecht, Germany) for 30 min to attach. A basic culture medium containing α-MEM, complemented with 15% fetal calf serum (FCS; HyClone, Logan, UT, USA), 100 U/mL penicillin, 2 mM l-glutamine (Biochrom), 1% amphotericin and 100 µg/mL streptomycin, was then carefully added to the flasks (standard culture conditions). Subsequently, the culture flasks were incubated at 5% carbon dioxide and 37 °C, and the culture medium was renewed three times per week. The cells were passaged after reaching a confluence of 80%. The cells of the first passage were split using the magnetic activated cell sorting (MACS) technique, linking anti-STRO-1 antibodies (SantaCruz, Santa Cruz, CA, USA) to STRO-1 surface receptors and using anti-PE microbead antibodies (Miltenyi Biotec, Bergisch Gladbach, Germany), according to the manufacturers’ directions. MACS-positive sorted cells, G-MSCs [20], were then dispersed to form colonies.

### 2.2. Colony-Forming Units (CFUs)

Under standard conditions, 1.63 cells/cm^2^ were cultivated. A colony was defined as 50 or more cells in a cluster. To demonstrate the CFUs, samples were fixed using 4% formalin and then stained with 0.1% crystal violet. The remaining G-MSCs forming single colonies were then isolated using cell scrapers, transferred to new 75 mL cell culture flasks and then grown under standard conditions.

### 2.3. Flowcytometric Analysis of Surface MSC Markers

To identify the MSCs’ predefined surface markers, 80% confluent G-MSCs were flowcytometrically analyzed [21]. The standard protocols for binding the primary antibodies and corresponding isotype controls were employed (Miltenyi Biotec). Afterwards, the results were assessed with a MACSQuant Flow Cytometry Analyzer (Miltenyi Biotec) and the analyzing software FlowJo 10 (Becton Dickinson).

### 2.4. Multilineage Differentiation of G-MSCs

To explore the potential of osteogenic differentiation, 2 × 10^4^ G-MSCs of the third passage were seeded on 6-well culture plates in an osteogenic inductive medium (PromoCell, Heidelberg, Germany). For the controls, identical G-MSC samples were grown at the same time in a basic culture medium. After two weeks, calcified sediments were stained with Alizarin Red (Sigma-Aldrich), and *runt-related transcription factor-2* (*RUNX*) and *alkaline phosphatase* (*ALP*) expressions were assessed using real-time polymerase chain reaction (PCR; LightCycler; Roche Life Science, Penzberg, Germany). To inspect the adipogenic potential of differentiation, 3 × 10^5^ third passage G-MSCs were grown on 6-well culture plates in an adipogenic inductive medium (PromoCell). For control, similar samples were cultured in a basic medium. The formation of cells containing lipid droplets was assessed using Oil Red O staining (Sigma-Aldrich), while *peroxisome proliferator-activated receptor gamma (PPARɣ)* and *lipoprotein lipase* (*LPL*) expressions were evaluated using PCR after 21 days. Chondrogenic differentiation was initiated by stimulating micro-masses of 3 × 10^4^ third passage G-MSCs with a chondrogenic inductive medium (PromoCell) in 6-well culture plates (Sarstedt). Similar samples of G-MSCs were cultured in a basic medium for control. At day 35, cell staining with Alcian Blue and Nuclear Fast Red as a counterstain (Sigma-Aldrich) was performed to investigate the produced glycosaminoglycans, and the expression of *aggrecan* (*ACAN*), as a cartilage-specific marker, was quantified. The inductive medium was renewed three times a week [1,22,23].

### 2.5. Thymoquinone Stimulation

The 3 × 10^5^ G-MSCs of the third passage were cultivated on 6-well culture plates under standard culture conditions. After attaining 80% confluence, 1 μg/mL of TQ (Sigma-Aldrich, Saint Louis, MS, USA) was supplemented for 24 h to the cells, while identical control samples were not stimulated. TQ stimulation was performed according to the described instructions and the recommended effective concentration in stem cells [16].

### 2.6. Flowcytometric Analysis of TLRs in G-MSCs after TQ Stimulation

The G-MSCs’ characterization using flowcytometry was performed before and after the TQ stimulation to determine changes in the expression of TLRs 1-10. For the staining of intracellular TLRs, the G-MSCs were initially fixed and permeabilized with a Fix & Perm kit (Cytofix/Cytoperm, BD Biosciences, Franklin Lakes, NJ, USA) before cell incubation. The following antibodies were used: anti-TLR1, anti-TLR3 and anti-TLR9 (all from Invitrogen, Waltham, MA, USA); anti-TLR2, anti-TLR4 and anti-TLR8 (all from Enzo Life Sciences, Lörrach, Germany); anti-TLR5 (R&D Systems, Hessen, Germany); anti-TLR6 (BD Life Sciences, East Rutherford, NJ, USA); and anti-TLR7 and anti-TLR10 (both from Invitrogen, Waltham, MA, USA). For the binding of the primary antibodies and their corresponding isotype controls, the standard protocols were followed, as described previously [1,3,9], and the results were then evaluated using the MACSQuant Flow Cytometry Analyzer (Miltenyi Biotec) and FlowJo 10 (Becton Dickinson, Franklin Lakes, NJ, USA).

### 2.7. Differential mRNA Expression of Pluripotency-Associated Genes after TQ Stimulation

To assess the expressed mRNA of pluripotency factors *sex-determining region Y-box 2 (SOX2)*, *homeobox protein Nanog (NANOG)*, *muscle segment homeobox (MSX-1)*, *octamer-binding transcription factor 4A (OCT4A)*, *REX* and *Krüppel-like factor 4 (KLF4)*, mRNA extraction and cDNA synthesis were performed for TQ-stimulated as well as unstimulated G-MSCs using the RNeasy Kit (Qiagen). Complementary cDNA was synthesized from 1–13 µL of RNA (1 µg/µL) using a reverse transcription (RT) kit (QuantiTect; Qiagen), with 1 µg of RNA put into reaction, according to the manufacturer’s instructions. The RT primer mix consisted of an optimized blend of oligo-dT and random primers dissolved in water, and RT was performed using incubation for 15 min at 42 °C. Quantitative real-time polymerase chain reaction (rt-PCR) was performed on a LightCycler 96 Real-Time PCR System (Roche Life Science) in a total volume of 20 µL using RealTime ready Catalog Assays (Roche Life Science), according to the manufacturer’s instructions. Using a 10 µL FastStart Essential DNA Probes Master (Roche Life Science) and a 5 µL specimen, cDNA.rt-PCR was performed, according to the manufacturer’s instructions [1,3], using temperature and time directions stated in the standard-qPCR protocol of LightCycler 96 for RealTime ready Assays (Roche Life Science). To select the most appropriate reference gene that would not be regulated by the stimulation, 19 reference genes were tested in stimulated and unstimulated G-MSCs. Data evaluation carried out using NormFinder software presented a stimulation regulation in most of the 19 tested reference genes and showed that *phosphoglycerate kinase 1 (PGK1)* would be the most appropriate gene to be applied as a reference gene in this study. Relative quantities were later normalized according to the expression of *PGK1* for each transcript. Relative quantification of the gene expression was performed using the 2−ΔΔCt method. All experiments were performed in triplicate and averaged. All primers (Table 2) were provided by Roche.

### 2.8. Statistical Analysis 

The Shapiro–Wilk test was applied to test for normality of the data. The data were not normally distributed. Differences between the stimulated (test) and unstimulated (control) groups were assessed using the nonparametric Wilcoxon signed-rank test (SPSS, version 11.5; IBM). The level of significance was set at *p* = 0.05.

## 3. Results

### 3.1. Phase Contrast Inverted Microscopy, CFUs and Analysis of Mesenchymal Stem Cells’ Predefined Surface Markers Using Flow Cytometry

**Figure 2:**
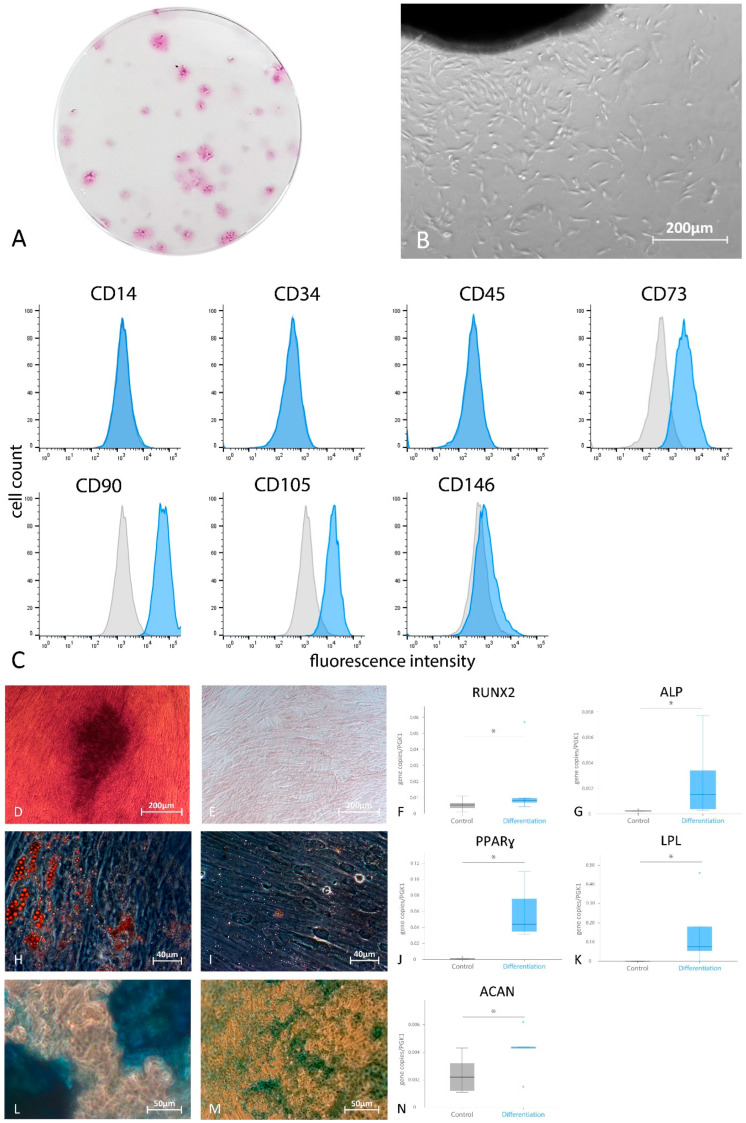
Microscopic appearance, colony formation, surface marker expression and differentiation potential of G-MSCs. (**A**) Crystal violet staining of G-MSCs’ colonies formed after 12 days and (**B**) microscopic appearance of the G-MSCs. (**C**) Flowcytometric expression profile of G-MSCs’ surface marker expression. Multilineage differentiation ability of G-MSCs. G-MSCs after stimulation with osteogenic inductive medium stained with Alizarin Red (**D**) and their controls (**E**) with the analysis of *RUNX* (**F**) and *ALP* (**G**) expressions. G-MSCs after stimulation with adipogenic differentiation medium stained with Oil Red O (**H**) and their controls (**I**) with the analysis of *PPARɣ* (**J**) and *LPL* (**K**) expressions. G-MSCs after stimulation with chondrogenic differentiation medium stained with Alcian Blue and Nuclear Fast Red (**L**) and their controls (**M**) with the analysis of *ACAN* (**N**). (*n* = 5; box-and-whisker plots with medians and quartiles; Wilcoxon signed-rank test, statistical significance marked with asterisk, *: *p* < 0.05). *ACAN*, aggrecan; *ALP*, alkaline phosphatase; *LPL*, lipoproteinlipase; *PPARɣ*, peroxisome proliferator-activated receptor-gamma; *RUNX*, runt-related transcription factor-2. Statistical outliers have been marked by blue dots.

After adherence of the free-gingival soft tissue pieces, cells spread out of them, creating adherent fibroblast-like groups. Twelve days later, the G-MSCs demonstrated CFUs (Figure 2A,B). Flowcytometric analysis of the G-MSCs’ predefined surface markers displayed an expression of CD73, CD90, CD105 and CD146, while CD14, CD34 and CD45 showed no expression (Figure 2C).

### 3.2. Multilineage Differentiation Potential of G-MSCs

After stimulation with the osteogenic medium, the G-MSCs displayed calcified deposits that were stained with Alizarin Red in distinction to their controls and significantly higher expressions of *RUNX* (median gene copies/PGK1copies, Q25/Q75) (0.0080, 0.0057/0.0333) and *ALP* (0.0015, 0.0003/0.0056) than their controls (*RUNX*: 0.0053, 0.0023/0.0088 and *ALP*: 0.0002, 0.0001/0.0003) (Figure 2D–G). Adding an adipogenic differentiation medium to the G-MSCs promoted the formation of cells containing lipid droplets stained with Oil-Red-O, in contrast to their controls, and they displayed significantly higher expressions of *PPARγ* (0.0440, 0.0335/0.0930) and *LPL* (0.0760, 0.0268/0.3200) than their controls (*PPARγ*: 0.0010, 0.0004/0.0022 and *LPL*: 0.0002, 0.0000/0.0003) (Figure 2H–K). Adding a chondrogenic differentiation medium to the G-MSCs initiated the formation of glycosaminoglycans that were stained with Alcian Blue, in contrast to their controls, and they displayed a significantly higher expression of *ACAN* (0.0043, 0.0029/0.0053) than their controls (0.0022, 0.0012/0.0038) (Figure 2L–N; *p* < 0.05, Wilcoxon signed-rank test).

### 3.3. Flowcytometric Analysis of TLRs in G-MSCs after Thymoquinone Stimulation

**Figure 3:**
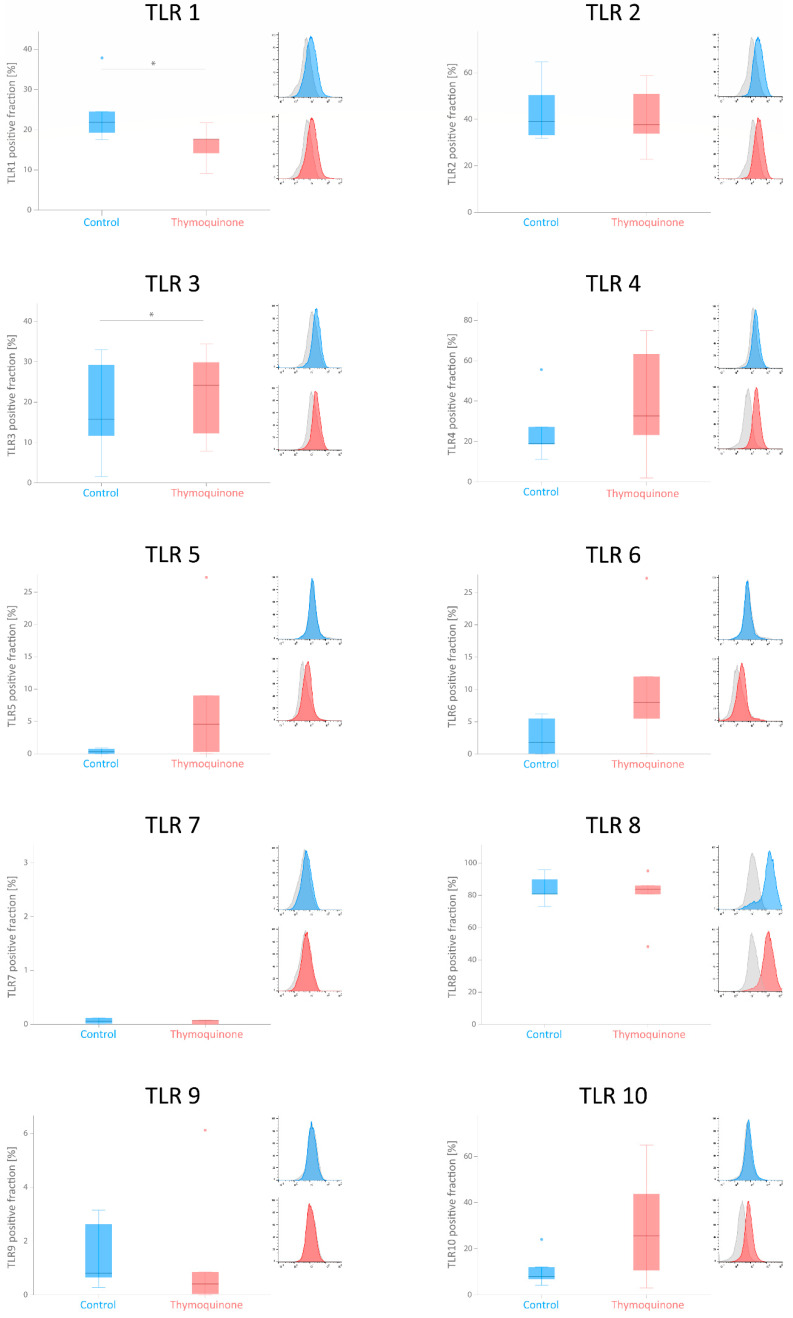
Flowcytometric percentage expression of TLRs 1–10 in G-MSCs with and without TQ stimulation. Expressed TLRs in G-MSCs (blue and red curves) and their isotype controls (grey curves) after incubation in a basic control medium and basic medium with TQ (*n* = 5; box-and-whisker plots with medians and quartiles). Significance at *p* < 0.05 marked with an asterisk * (Wilcoxon signed-rank test). Statistical outliers have been marked by blue and red dots.

Stimulation with TQ modulated TLR expression, as seen in Table 3. Stimulated G-MSCs showed a significantly lower expression of TLR1 and higher expression of TLR3 than those of the control group on the protein level (Table 3 and Figure 3).

### 3.4. Differential mRNA Expression of Pluripotency-Associated Genes in G-MSCs following TQ Stimulation

**Figure 4:**
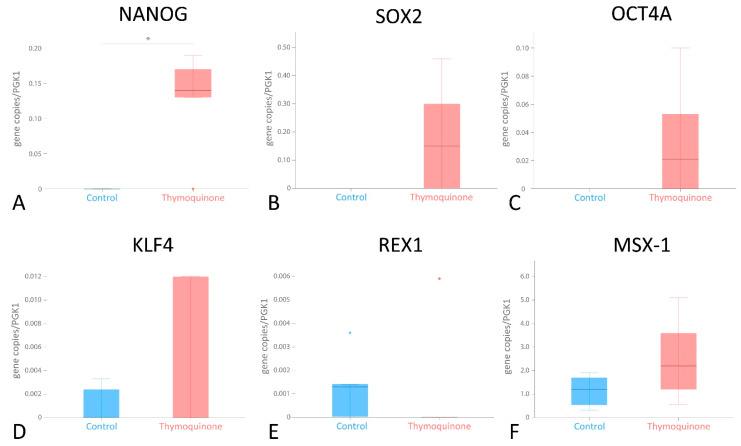
mRNA expression of pluripotency factors after TQ stimulation. Changes in mRNA expression of the stemness-associated factors (**A**) homeobox protein *Nanog* (*NANOG*), (**B**) *sex-determining region Y (SRY)-box 2* (*SOX2*), (**C**) *octamer-binding transcription factor 4* (*OCT4A*), (**D**) *Krüppel-like factor 4* (*KLF4*), (**E**) *REX1* and (**F**) *muscle segment homeobox* (*MSX-1*) after G-MSC stimulation with TQ compared to the unstimulated control group. Significance at *p* < 0.05 marked with an asterisk * (Wilcoxon signed-rank test). Statistical outliers have been marked by blue and red dots.

Stimulation with TQ non-significantly elevated the expression of most investigated genes. A significantly higher expression of *NANOG* (0.14, 0.06/0.18) (*p* < 0.05, Wilcoxon signed-rank test) than that of the control group (0.00, 0.00/0.00) was observed following TQ stimulation (Table 4 and Figure 4).

## 4. Discussion

TLR expression and activation are strongly associated with various inflammatory conditions, including periodontitis [1,22,23,24]. A controlled or targeted expression and ligation of specific TLRs are suggested to modify MSCs’ migration, differentiation, survival, proliferation and immunomodulation, as well as regenerative abilities and stemness-associated pluripotency factors [1,3,22,25,26,27]. Previous studies proposed that MSC-associated TLR expression profiles and MSC pluripotency factors play a crucial role in defining the biological healing processes and are chiefly affected by the source of the expressing cells, as well as the surrounding microenvironmental influences, such as inflammation [1,3,28,29,30]. Among these modulating incentives, bioactive molecules from natural products have gained an increased attention in recent years due to their chemical diversity as promising and effective drug candidates while being accessible and affordable [11,15,29,31]. TQ, the natural bioactive component of NS seeds, has been expansively reviewed in many studies and displayed numerous medicinal activities with antimicrobial, anti-inflammatory, antioxidant and immune-regulating effects [11,32]. Among its described properties, TQ exhibited MSC-modulating functions, endorsing a high therapeutic and regenerative value in many diseases, such as periodontitis [11]. As explained in previous studies [17], TQ promoted *c-MET* and *CXCR4* signaling pathways, encouraging MSC migration. Furthermore, it demonstrated the ability to alter the immunomodulatory potential and TLR expression in MSCs, as well as their stemness, by regulating the associated pluripotency gene expression in vitro and in vivo [16,17]. Other investigations described the increased healing ability via MSCs after TQ administration to damaged sites [19,33]. In the present investigation, the TQ-induced modulation of TLR expression and pluripotency factors of G-MSCs was explored for the first time.

Similar to previous studies, anti-STRO-1 antibodies were used for G-MSC isolation. The G-MSCs showed positive expressions of CD90, CD105 and CD73 while being negative for CD14, CD34 and CD45 [1,20,21]. The G-MSCs showed an ability to form CFUs, in addition to a notable multilineage potential for differentiation into bone, adipose and cartilaginous tissues.

Analogous to former investigations, G-MSCs expressed a similar profile of TLRs [1,3,7] and pluripotency factors [34,35,36] and were subsequently stimulated by TQ, as described earlier. TQ stimulation of G-MSCs significantly increased the expression of TLR3, with a simultaneous significant suppression of TLR1. Non-significant upregulation of TLRs 4, 5, 6, 7, 8 and 10, along with a decline in TLRs 2 and 9, was also observed. This effect was similarly reported in earlier studies on bone marrow mesenchymal stem cells (BM-MSCs) under different inflammatory conditions with a TQ-mediated increase in TLR3 and TLR4 expressions [16], along with the suppression of TLR2 [37,38,39]. This detected immunomodulation via TQ might be explained by its regulatory mechanism on TLR pathway components, as well as their downstream effectors, as reported with nuclear *factor kappa-light-chain-enhancer of activated B cells* (*NF-κB*), *interferon regulatory transcription factor* *3* (*IRF-3*) and *phosphoinositide 3-kinases* (*PI3Ks*) in various clinical and experimental settings [37,38,40,41]. This distinct TQ-mediated modulation of G-MSCs’ TLRs could influence the G-MSCs’ therapeutic potential in vivo. Through an increased expression of TLRs 4, 6, 7, 8 and 10, an early cellular activation might be attained. This would allow us to identify important microbial components, such as lipopolysaccharides (LPS) and lipoproteins, which are primarily involved in the development of periodontitis [42] and other inflammatory diseases [43]. On the other hand, it has been reported in previous studies [44] that the progression of different in vivo inflammatory reactions can be regulated by TLR4. High TLR4 expression can quickly trigger *mitogen-activated protein kinase* (*MAPK*) and *NF-κB* via the pathway of the *myeloid differentiation primary response 88* (*MYD88*), which promotes the release of pro-inflammatory cytokines [45]. Furthermore, the increased TLR4 expression endorses an increased binding of TLR4 to its ligands, thus encouraging cellular and humoral immunity, which could alleviate the progression of the inflammation [44].

More importantly, the detected significant decrease in TLR1 and increase in TLR3 expression following TQ stimulation could play an important role in the therapeutic value of G-MSCs. As perceived in previous studies, TLR3, which serves mainly as a viral RNA detector, has another important function in the recognition of damage-associated molecular patterns (DAMPS) as primary danger signals in periodontal pathogenesis [46]. Although periodontal diseases are infectious diseases triggered by oral microorganisms, in certain conditions, DAMPs might induce inflammatory responses before host cells identify pathogen-associated molecular patterns, leading to earlier development and prolongation of periodontal disease [46,47]. By significantly increasing TLR3 expression with TQ modulation in G-MSCs, an elevated TLR3 priming by DAMPS [46] is facilitated to detect primary signals of periodontal disease [46] and to promote a cell cycle transition of G-MSCs into stages of differentiation and maturation, shifting the cells into a highly osteogenic phenotype [1,48] that is pivotal for regenerative periodontal therapy [49]. Moreover, it can direct the G-MSCs towards the G-MSC2 anti-inflammatory phenotype with an increased *indoleamine-pyrrole 2,3-dioxygenase* (*IDO*) secretion, which exerts an important immunosuppressive role in periodontal therapeutic processes [3,50]. Furthermore, a concurrent decrease in TLR1 expression endorses the same therapeutic effect, leading to reduced inflammatory cytokine release and related periodontal bone loss [42].

Pluripotency describes a cell’s ability to give rise to differentiated derivatives representing each of the three primary germ layers [51]. Previous investigations have identified a number of pluripotency transcription factors, which promote the pluripotent capacity and are associated with an increased stemness of different types of stem/progenitor cells, including G-MSCs [1,35]. These factors can modulate stem/progenitor cells’ pluripotency and differentiation through polycomb repressive complexes (PRC) and microRNAs, as well as the cooperation with each other in the epigenetic and transcriptional regulation of chief stem cell genes [51]. Further studies have shown a significant modulation of pluripotency factors after priming by different microenvironmental inducements. This effect was explained by their regulation of signaling networks [1,52,53], cell cycle genes and different effector molecules [54]. Among these stimulating factors, herbal and natural products have displayed promising results in stemness regulation for better therapeutic outcomes [13,55,56]. Considering its outstanding effects as a natural compound, TQ was tested in multiple stem cell investigations to regulate regeneration and pluripotency, and it displayed significant modulative effects [16,17,39,57]. In the current study, TQ significantly promoted G-MSC expression of *NANOG* and non-significantly promoted that of all other investigated pluripotency factors, except *REX1* and *KLF4*. This significant increase in *NANOG*, as a critical pluripotency gene for stem cell regulation [58], emphasizes the TQ-mediated effect on G-MSCs for the maintenance of their pluripotency [59]. Although previous studies reported similar effects of TQ on pluripotency genes in BM-MSCs [11,16], other investigations on cancer stem cells described a decreased stemness and pluripotency after TQ therapeutic applications, giving it potential therapeutic abilities in cancer treatment [57,60,61].

**Figure 5:**
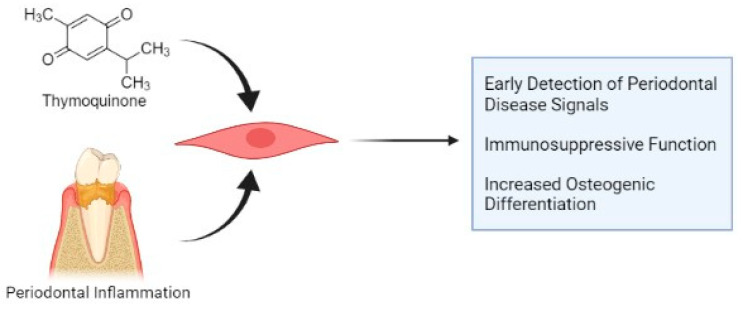
Potential thymoquinone-mediated modulation of gingival mesenchymal stem/progenitor cells (G-MSCs) during periodontal inflammation (created with BioRender.com; accessed on 9 April 2022).

This detected TQ-mediated interplay of G-MSCs’ TLR expression and the pluripotency factor *NANOG* could affect G-MSCs’ therapeutic properties. While maintaining pluripotency through increased *NANOG* expression in non-inflammatory conditions to protect the cells from senescence [62], the inflammatory microenvironment and DAMPS in periodontal diseases can increasingly prime the TQ-endorsed TLR3 [46]. This would subsequently lead to the previously described transition of G-MSCs from a primary stage of higher pluripotency into a stage of maturation in the cells’ cycle, with a high osteogenic potential for periodontal repair [1,48], immunosuppressive functions counteracting the inflammation and an earlier detection of periodontal disease onset [3,46] (Figure 5).

The following limitations have to be acknowledged in the current investigation: First, this is a primary-stage in vitro study investigating the potential effect of TQ on G-MSC TLR expression, pluripotency and differentiation potential in inflammatory conditions, such as periodontitis. This study therefore explains several points on a hypothetical basis and would certainly benefit from the planned future investigations to discover the exact mechanisms of the current outcomes. Analyzing co-variables, such as interacting factors of the innate or acquired immune response and interplay between TQ, TLRs and pluripotency factors during differentiation, and performing the tests in an inflammatory in vitro milieu, as well as possible clinical studies, may play a major role in extending our knowledge about the modulating effect of TQ on G-MSCs. Moreover, the present study examined a relatively small sample size similar to other related studies (1, 3]. Increasing the sample size might have an effect on the performed statistical evaluation and increase the number of obtained significances [63].

Within these limitations, the outcome of the current study provides important insight into the several effects of TQ on G-MSCs. TQ priming of G-MSCs during periodontal treatment might positively affect the cells’ therapeutic properties through their biomodulation. Nevertheless, this topic needs further elaboration and expansion, especially on a clinical level, to confirm the suggested therapeutic value.

## Figures and Tables

**Table 1:** Individual data of the study donors.

Donor	Age	Gender
1	26	Female
2	24	Female
3	41	Female
4	87	Male
5	23	Male

**Table 2:** Primers used for real-time PCR and their IDs.

Assay ID	Gene Symbol	Accession ID
113380	*RUNX2 Homo sapiens*	ENST00000359524
110607	*PPARɣ Homo sapiens*	ENST00000287820
103448	*ALP Homo sapiens*	ENST00000374840
138057	*ACAN Homo sapiens*	ENST00000439576
113230	*LPL Homo sapiens*	ENST00000311322
148147	*Nanog Homo sapiens*	ENST00000229307
137021	*MSX1* *Homo sapiens*	ENST00000382723
111867	*Sox2 Homo sapiens*	ENST00000325404
125775	*KLF4* *Homo sapiens*	ENST00000358094
122584	*REXO1* *Homo sapiens*	ENST00000170168
113034	*Oct4A Homo sapiens*	ENST00000259915
102083	*PGK1 Homo sapiens*	ENST00000373316

**Table 3:** TQ stimulation for 24 h and corresponding TLR protein expression using flowcytometry (median expression percentage, Q25/Q75); significance at *p* < 0.05 (Wilcoxon signed-rank test).

Toll-like Receptor	Unstimulated G-MSCs (Control)	1 µg/mL TQ-Stimulated G-MSCs	*p*-Value
TLR 1	21.8163, 18.3802/31.1601	17.6136, 11.6273/19.6647	0.043
TLR 2	39.0383, 32.4885/57.5765	37.6595, 28.2930/54.8602	>0.05
TLR 3	15.7353, 6.6038/31.1184	24.1686, 10.0862/32.1546	0.043
TLR 4	18.9917, 14.8641/42.0839	32.6764, 12.5569/69.0827	>0.05
TLR 5	0.3003, 0.0033/0.8241	4.8510, 0.1363/18.1387	>0.05
TLR 6	1.8360, 0.0330/5.8871	8.0203, 2.7385/19.6100	>0.05
TLR 7	0.0575, 0. 0067/8.6946	0.0656, 0.0000/1.9158	>0.05
TLR 8	80.8476, 76.8108/92.7699	83.7606, 64.4336/90.5349	>0.05
TLR 9	0.8045, 0.4643/2.8881	0.4037, 0.0160/3.4859	>0.05
TLR 10	7.9431, 5.5215/18.0287	25.6538, 6.8429/54.3215	>0.05

**Table 4:** TQ stimulation and corresponding mRNA expression of pluripotency-associated genes (median gene copies/PGK1 copies, Q25/Q75).

Stemness Transcriptional Factor	Unstimulated G-MSCs (Control)	1 µg/mL TQ-Stimulated G-MSCs	*p*-Value
*NANOG*	No expression	0.1400, 0.0650/0.1800	0.043
*SOX2*	No expression	0.1500, 0.0004/0.3800	>0.05
*OCT4*	No expression	0.0210, 0.0000/0.0765	>0.05
*MSX1*	1.2000, 0.4200/1.8000	2.2000, 0.8750/4.3500	>0.05
*REX1*	0.0013, 0.0025/0.0000	0.0000, 0.0000/0.0030	>0.05
*KLF4*	0.0000, 0.0000/0.0029	0.0000, 0.0000/0.0120	>0.05

## Data Availability

The data presented in this study are available on request from the corresponding author.

## References

[1] Mekhemar M., Tolle J., Dorfer C., Fawzy El-Sayed K. (2020). Gingival Mesenchymal Stem/Progenitor Cells. J. Clin. Periodontol..

[2] Fawzy El-Sayed K.M., Dorfer C.E. (2016). Gingival Mesenchymal Stem/Progenitor Cells: A Unique Tissue Engineering Gem. Stem Cells Int..

[3] Mekhemar M.K., Adam-Klages S., Kabelitz D., Dörfer C.E., Fawzy El-Sayed K.M. (2018). TLR-induced immunomodulatory cytokine expression by human gingival stem/progenitor cells. Cell Immunol..

[4] Fawzy El-Sayed K., Graetz C., Kohnlein T., Mekhemar M., Dorfer C. (2018). Effect of total sonicated Aggregatibacter actinomycetemcomitans fragments on gingival stem/progenitor cells. Med. Oral Patol. Oral Cir. Bucal..

[5] Fawzy El-Sayed K.M., Paris S., Becker S.T., Neuschl M., De Buhr W., Salzer S., Wulff A., Elrefai M., Darhous M.S., El-Masry M. (2012). Periodontal regeneration employing gingival margin-derived stem/progenitor cells: An animal study. J. Clin. Periodontol..

[6] Fawzy El-Sayed K.M., Elahmady M., Adawi Z., Aboushadi N., Elnaggar A., Eid M., Hamdy N., Sanaa D., Dorfer C.E. (2019). The periodontal stem/progenitor cell inflammatory-regenerative cross talk: A new perspective. J. Periodontal. Res..

[7] Fawzy-El-Sayed K., Mekhemar M., Adam-Klages S., Kabelitz D., Dorfer C. (2016). TlR expression profile of human gingival margin-derived stem progenitor cells. Med. Oral Patol. Oral Cir. Buccal.

[8] Fawzy El-Sayed K.M., Boeckler J., Dörfer C.E. (2017). TLR expression profile of human alveolar bone proper-derived stem/progenitor cells and osteoblasts. J. Craniomaxillofac. Surg..

[9] Fawzy El-Sayed K.M., Klingebiel P., Dörfer C.E. (2016). Toll-like Receptor Expression Profile of Human Dental Pulp Stem/Progenitor Cells. J. Endod..

[10] Zhou L., Dorfer C.E., Chen L., Fawzy El-Sayed K.M. (2017). Porphyromonas gingivalis lipopolysaccharides affect gingival stem/progenitor cells attributes through NF-kappaB, but not Wnt/beta-catenin, pathway. J. Clin. Periodontol..

[11] Mekhemar M., Hassan Y., Dörfer C. (2020). Nigella sativa and Thymoquinone: A Natural Blessing for Periodontal Therapy. Antioxidants.

[12] WHO (2013). Traditional Medicine Strategy: 2014–2023.

[13] Udalamaththa V.L., Jayasinghe C.D., Udagama P.V. (2016). Potential role of herbal remedies in stem cell therapy: Proliferation and differentiation of human mesenchymal stromal cells. Stem Cell Res. Ther..

[14] Johnson T.C., Siegel D. (2017). Directing Stem Cell Fate: The Synthetic Natural Product Connection. Chem. Rev..

[15] Mekhemar M., Geib M., Kumar M., Radha Hassan Y., Dorfer C. (2021). *Salvadora persica*: Nature’s Gift for Periodontal Health. Antioxidants.

[16] Alimoradi E., Sisakhtnezhad S., Akrami H. (2018). Thymoquinone influences the expression of genes involved in self-renewal and immunomodulatory potential of mouse bone marrow-derived mesenchymal stem cells in vitro. Environ. Toxicol. Pharmacol..

[17] Rezaei N., Sardarzadeh T., Sisakhtnezhad S. (2020). Thymoquinone promotes mouse mesenchymal stem cells migration in vitro and induces their immunogenicity in vivo. Toxicol. Appl. Pharmacol..

[18] Arslan A.H., Tomruk C.Ö., Meydanlı E.G., Özdemir İ., Duygu Çapar G., Kütan E., Yilmaz A., Ülker G.M.Y. (2017). Histopathological evaluation of the effect of systemic thymoquinone administration on healing of bone defects in rat tibia. Biotechnol. Biotechnol. Equip..

[19] Radwan R.R., Mohamed H.A. (2018). Nigella sativa oil modulates the therapeutic efficacy of mesenchymal stem cells against liver injury in irradiated rats. J. Photochem. Photobiol. B Biol..

[20] El-Sayed K.M., Paris S., Graetz C., Kassem N., Mekhemar M., Ungefroren H., Fandrich F., Dorfer C. (2015). Isolation and characterisation of human gingival margin-derived STRO-1/MACS(+) and MACS(-) cell populations. Int. J. Oral Sci..

[21] Dominici M., Le Blanc K., Mueller I., Slaper-Cortenbach I., Marini F., Krause D., Deans R., Keating A., Prockop D., Horwitz E. (2006). Minimal criteria for defining multipotent mesenchymal stromal cells. The International Society for Cellular Therapy position statement. Cytotherapy.

[22] Delarosa O., Dalemans W., Lombardo E. (2012). Toll-like receptors as modulators of mesenchymal stem cells. Front Immunol..

[23] Hans M., Hans V.M. (2011). Toll-like receptors and their dual role in periodontitis: A review. J. Oral Sci..

[24] Lu Y., Li X., Liu S., Zhang Y., Zhang D. (2018). Toll-like Receptors and Inflammatory Bowel Disease. Front Immunol..

[25] Castro-Manrreza M.E., Montesinos J.J. (2015). Immunoregulation by Mesenchymal Stem Cells: Biological Aspects and Clinical Applications. J. Immunol. Res..

[26] Najar M., Krayem M., Meuleman N., Bron D., Lagneaux L. (2017). Mesenchymal Stromal Cells and Toll-Like Receptor Priming: A Critical Review. Immune Netw..

[27] Zhang L., Liu D., Pu D., Wang Y., Li L., He Y., Li Y., Li L., Qiu Z., Zhao S. (2015). The role of Toll-like receptor 3 and 4 in regulating the function of mesenchymal stem cells isolated from umbilical cord. Int. J. Mol. Med..

[28] Kadle R.L., Abdou S.A., Villarreal-Ponce A.P., Soares M.A., Sultan D.L., David J.A., Massie J., Rifkin W.J., Rabbani P., Ceradini D.J. (2018). Microenvironmental cues enhance mesenchymal stem cell-mediated immunomodulation and regulatory T-cell expansion. PLoS ONE..

[29] Nembo E.N., Hescheler J., Nguemo F. (2020). Stem cells in natural product and medicinal plant drug discovery—An overview of new screening approaches. Biomed. Pharmacother..

[30] Yamamoto T., Ugawa Y., Kawamura M., Yamashiro K., Kochi S., Ideguchi H., Takashiba S. (2018). Modulation of microenvironment for controlling the fate of periodontal ligament cells: The role of Rho/ROCK signaling and cytoskeletal dynamics. J. Cell Commun. Signal..

[31] Rahman M.A., Saha S.K., Rahman M.S., Uddin M.J., Uddin M.S., Pang M.G., Rhim H., Cho S.-G. (2020). Molecular Insights into Therapeutic Potential of Autophagy Modulation by Natural Products for Cancer Stem Cells. Front. Cell. Dev. Biol..

[32] Yimer E.M., Tuem K.B., Karim A., Ur-Rehman N., Anwar F. (2019). *Nigella sativa* L. (Black Cumin): A Promising Natural Remedy for Wide Range of Illnesses. Evid. Based Complement. Alternat. Med..

[33] Sallehuddin N., Nordin A., Bt Hj Idrus R., Fauzi M.B. (2020). *Nigella sativa* and Its Active Compound, Thymoquinone, Accelerate Wound Healing in an In Vivo Animal Model: A Comprehensive Review. Int. J. Environ. Res. Public Health.

[34] Fawzy El-Sayed K.M., Hein D., Dörfer C.E. (2019). Retinol/inflammation affect stemness and differentiation potential of gingival stem/progenitor cells via Wnt/β-catenin. J. Periodontal. Res..

[35] Fawzy El-Sayed K.M., Bittner A., Schlicht K., Mekhemar M., Enthammer K., Höppner M., Es-Souni M., Schulz J., Laudes M., Graetz C. (2021). Ascorbic Acid/Retinol and/or Inflammatory Stimuli’s Effect on Proliferation/Differentiation Properties and Transcriptomics of Gingival Stem/Progenitor Cells. Cells.

[36] Fawzy El-Sayed K.M., Nguyen N., Dorfer C.E. (2020). Ascorbic Acid, Inflammatory Cytokines (IL-1beta/TNF-alpha/IFN-gamma), or Their Combination’s Effect on Stemness, Proliferation, and Differentiation of Gingival Mesenchymal Stem/Progenitor Cells. Stem Cells Int..

[37] Arjumand S., Shahzad M., Shabbir A., Yousaf M.Z. (2019). Thymoquinone attenuates rheumatoid arthritis by downregulating TLR2, TLR4, TNF-α, IL-1, and NFκB expression levels. Biomed. Pharmacother..

[38] Abulfadl Y.S., El-Maraghy N.N., Ahmed A.E., Nofal S., Abdel-Mottaleb Y., Badary O.A. (2018). Thymoquinone alleviates the experimentally induced Alzheimer’s disease inflammation by modulation of TLRs signaling. Hum. Exp. Toxicol..

[39] Kalamegam G., Alfakeeh S.M., Bahmaid A.O., AlHuwait E.A., Gari M.A., Abbas M.M., Ahmed F., Abu-Elmagd M., Pushparaj P.N. (2020). In Vitro Evaluation of the Anti-inflammatory Effects of Thymoquinone in Osteoarthritis and In Silico Analysis of Inter-Related Pathways in Age-Related Degenerative Diseases. Front. Cell Dev. Biol..

[40] Aziz N., Son Y.-J., Cho J.Y. (2018). Thymoquinone Suppresses IRF-3-Mediated Expression of Type I Interferons via Suppression of TBK1. Int. J. Mol. Sci..

[41] Xu D., Ma Y., Zhao B., Li S., Zhang Y., Pan S., Wu Y., Wang J., Wang D., Pan H. (2014). Thymoquinone induces G2/M arrest, inactivates PI3K/Akt and nuclear factor-κB pathways in human cholangiocarcinomas both in vitro and in vivo. Oncol. Rep..

[42] Song B., Zhang Y.L., Chen L.J., Zhou T., Huang W.K., Zhou X., Shao L.Q. (2017). The role of Toll-like receptors in periodontitis. Oral Dis..

[43] Ghosh S.S., Wang J., Yannie P.J., Ghosh S. (2020). Intestinal Barrier Dysfunction, LPS Translocation, and Disease Development. J. Endocr. Soc..

[44] Qi W., Yang X., Ye N., Li S., Han Q., Huang J., Wu B. (2019). TLR4 gene in the regulation of periodontitis and its molecular mechanism. Exp. Ther. Med..

[45] Zhang X.Y., Liu Y., He T., Yang T.T., Wu J., Cianflone K., Lu H.-L. (2018). Anaphylatoxin C5a induces inflammation and reduces insulin sensitivity by activating TLR4/NF-kB/PI3K signaling pathway in 3T3-L1 adipocytes. Biomed. Pharmacother..

[46] Mori K., Yanagita M., Hasegawa S., Kubota M., Yamashita M., Yamada S., Kitamura M., Murakami S. (2015). Necrosis-induced TLR3 Activation Promotes TLR2 Expression in Gingival Cells. J. Dent. Res..

[47] Marchesan J.T., Girnary M.S., Moss K., Monaghan E.T., Egnatz G.J., Jiao Y., Zhang S., Beck J., Swanson K.V. (2020). Role of inflammasomes in the pathogenesis of periodontal disease and therapeutics. Periodontology 2000.

[48] Zhu Y., Li Q., Zhou Y., Li W. (2019). TLR activation inhibits the osteogenic potential of human periodontal ligament stem cells through Akt signaling in a Myd88- or TRIF-dependent manner. J. Periodontol..

[49] Alvarez R., Lee H.-L., Wang C.-Y., Hong C. (2015). Characterization of the osteogenic potential of mesenchymal stem cells from human periodontal ligament based on cell surface markers. Int. J. Oral Sci..

[50] Chaikeawkaew D., Everts V., Pavasant P. (2020). TLR3 activation modulates immunomodulatory properties of human periodontal ligament cells. J. Periodontol..

[51] Tsai C.C., Hung S.C. (2012). Functional roles of pluripotency transcription factors in mesenchymal stem cells. Cell Cycle.

[52] Hawkins K., Joy S., McKay T. (2014). Cell signalling pathways underlying induced pluripotent stem cell reprogramming. World J. Stem Cells.

[53] Weidgang C.E., Seufferlein T., Kleger A., Mueller M. (2016). Pluripotency Factors on Their Lineage Move. Stem Cells Int..

[54] Noronha N.d.C., Mizukami A., Caliári-Oliveira C., Cominal J.G., Rocha J.L.M., Covas D.T., Swiech K., Malmegrim K.C.R. (2019). Priming approaches to improve the efficacy of mesenchymal stromal cell-based therapies. Stem Cell Res. Ther..

[55] Abruzzo P.M., Canaider S., Pizzuti V., Pampanella L., Casadei R., Facchin F., Ventura C. (2020). Herb-Derived Products: Natural Tools to Delay and Counteract Stem Cell Senescence. Stem Cells Int..

[56] Vizetto-Duarte C., Castelo-Branco P., Custódio L. (2021). Marine Natural Products as a Promising Source of Therapeutic Compounds to Target Cancer Stem Cells. Curr. Med. Chem..

[57] Fatfat Z., Fatfat M., Gali-Muhtasib H. (2021). Therapeutic potential of thymoquinone in combination therapy against cancer and cancer stem cells. World J. Clin. Oncol..

[58] Yu P., Nie Q., Tang C., Zhang L. (2018). Nanog induced intermediate state in regulating stem cell differentiation and reprogramming. BMC Syst. Biol..

[59] Chen L., Tong Q., Chen X., Jiang P., Yu H., Zhao Q., Sun L., Liu C., Gu B., Zheng Y. (2021). PHC1 maintains pluripotency by organizing genome-wide chromatin interactions of the Nanog locus. Nat. Commun..

[60] Liou Y.F., Chen P.N., Chu S.C., Kao S.H., Chang Y.Z., Hsieh Y.S., Chang H.-R. (2019). Thymoquinone suppresses the proliferation of renal cell carcinoma cells via reactive oxygen species-induced apoptosis and reduces cell stemness. Environ. Toxicol..

[61] Bhattacharjee M., Upadhyay P., Sarker S., Basu A., Das S., Ghosh A., Ghosh S., Adhikary A. (2020). Combinatorial therapy of Thymoquinone and Emodin synergistically enhances apoptosis, attenuates cell migration and reduces stemness efficiently in breast cancer. Biochim. Biophys. Acta Gen. Subj..

[62] Chang C.C., Li H.H., Tsou S.H., Hung H.C., Liu G.Y., Korolenko T.A., Lai T.-J., Ho Y.-J., Lin C.-L. (2020). The Pluripotency Factor Nanog Protects against Neuronal Amyloid β-Induced Toxicity and Oxidative Stress through Insulin Sensitivity Restoration. Cells.

[63] Serdar C.C., Cihan M., Yücel D., Serdar M.A. (2021). Sample size, power and effect size revisited: Simplified and practical approaches in pre-clinical, clinical and laboratory studies. Biochem. Med..

